# Combining clinical and left atrial electromechanical remodelling data: potential to improve atrial fibrillation ablation outcome prediction

**DOI:** 10.1186/s12911-025-03200-7

**Published:** 2025-09-29

**Authors:** Neil Bodagh, Iain Sim, Mahta Haghighat Ghahfarokhi, Kyaw Soe Tun, Irum Kotadia, Magda Klis, Vinush Vigneswaran, Jose Alonso Solis Lemus, John Whitaker, Ali Gharaviri, Pier-Giorgio Masci, Amedeo Chiribiri, Steven Niederer, Mark O’Neill, Steven E. Williams

**Affiliations:** 1 Guy’s and St Thomas’ Hospital & King’s College London, Westminster Bridge Road, London, SE1 7EH UK; 2 University of Edinburgh, Edinburgh, EH8 9YL UK; 3 Imperial College London, London, W12 0NN UK; 4 King’s College London, London, UK

**Keywords:** Atrial fibrillation, Catheter ablation, Electromechanical remodelling, Outcome prediction, Electroanatomic mapping

## Abstract

**Background:**

Reliable outcome prediction following atrial fibrillation (AF) catheter ablation is important to inform shared decision-making. The extent of atrial electromechanical remodelling can be determined through magnetic resonance imaging and electroanatomic mapping data analysis. Combining these data with clinical data could improve the accuracy of outcome prediction models.

**Objective:**

To investigate how left atrial electromechanical remodelling data can be utilised to predict outcomes for first-time and repeat AF ablation.

**Methods:**

A retrospective analysis of 123 patients undergoing first-time ablation was conducted. Clinical, imaging and electroanatomic mapping variables associated with arrhythmia recurrence were identified using univariable logistic regression and combined into a multivariable model. Predictive ability for treatment response was examined using receiver-operator characteristic curve, time-to-event analyses and compared to pre-existing clinical risk scores.

**Results:**

A multivariable model comprising age, weight, hypertension, left atrial ejection fraction and mean left atrial voltage attained a c-statistic of 0.733 (95% CI 0.545–0.894) for predicting arrhythmia recurrence after one procedure, and 0.680 (95% CI 0.509–0.852) for repeat ablation. Kaplan-Meier analysis demonstrated a higher rate of arrhythmia recurrence amongst patients identified as high-risk (log-rank *p* = 0.010). Amongst pre-existing risk scores, CAAP-AF had the highest predictive value for predicting index procedure response (AUC 0.653, 95% CI 0.527–0.779).

**Conclusion:**

The model developed in this study demonstrated the potential for improved index AF ablation outcome prediction accuracy compared to pre-existing risk scores. Combined models integrating data measuring the extent of atrial electromechanical remodelling could optimise patient selection for repeat ablation, offering the potential to improve outcomes and reduce the volume of unnecessary procedures.

**Supplementary Information:**

The online version contains supplementary material available at 10.1186/s12911-025-03200-7.

## Introduction

Atrial fibrillation (AF) recurs following an index catheter ablation procedure in 15–40% of patients with paroxysmal AF and 30–60% of patients with persistent AF [1, 2]. Following repeat ablation, atrial fibrillation or tachycardia recurs in approximately 25–45% of patients [3, 4]. Several clinical risk scores have been developed to identify patients at higher risk of arrhythmia recurrence following ablation to facilitate shared decision making [5]. Whilst clinical risk scores have shown promise in single-centre studies, most scores perform poorly in validation cohorts [6], particularly in predicting the outcome of repeat ablation [7]. The reasons for this are unclear but may reflect that currently available clinical scores most often comprise a combination of clinical and echocardiographic parameters. Whilst such parameters are readily obtainable, they are limited in their ability to characterise atrial electromechanical perturbations conducive to arrhythmia recurrence.

Alternatively, measurement of the extent of atrial electromechanical remodelling might improve treatment response prediction. Atrial cardiac magnetic resonance imaging enables comprehensive characterisation of atrial size, shape, and contractile function through the measurement of atrial area, volume and ejection fraction, while late gadolinium enhancement imaging shows potential for determining the extent of atrial fibrosis [8]. Electroanatomic mapping data are routinely collected during ablation procedures and provide detailed characterisation of electrophysiological function [9]. Specifically, aberrations in parameters such as voltage, conduction velocity, and electrogram duration may reflect electrophysiological perturbations within atrial tissue that facilitate arrhythmia initiation and/or maintenance. We hypothesised that magnetic resonance imaging and electroanatomic mapping data could be combined to quantify the extent of atrial electromechanical remodelling and more accurately predict ablation outcomes.

In this study, we aimed to determine whether a predictive model incorporating left atrial electromechanical remodelling data could enhance the prediction of ablation outcomes compared to pre-existing clinical risk scores for both first-time and repeat AF ablation procedures.

## Methods

### Patient population

A retrospective analysis of consecutive patients attending for first-time AF ablation from 1st January 2016 to 31st December 2018 was undertaken. Patients were included if pre-procedural cardiac magnetic resonance imaging was performed and intra-procedural electroanatomic maps were created during coronary sinus pacing. Patients were excluded if there was a history of congenital heart disease, previous cardiac surgery, previous left atrial ablation, magnetic resonance image quality was inadequate or electroanatomic maps did not meet predefined quality metrics as previously described [8]. The study was performed in accordance with the Declaration of Helsinki. The data were collected during routine care. The requirements for ethical approval and written consent were waived following UK Health Research Authority review (18/HRA/0083).

### Data collection and risk score calculation

Pre-ablation clinical data and follow-up data were documented for all patients. Clinical risk scores, defined as a combination of two or more clinical and/or echocardiographic parameters within a statistical model to predict the likelihood of arrhythmia recurrence following catheter ablation, were identified from a previously published systematic review [5]. The original search strategy, fully detailed in the previous publication, used a combination of AF-related text and index terms, with the ‘model’ component informed by a previously validated search filter strategy [10]. Using this search strategy, we repeated the searches from database inception to 12 June 2024 to ensure inclusion of subsequently published scores which post-date the previous systematic review.

The following AF clinical risk scores were calculated in patients who had the full set of parameters necessary for risk score calculation available (*n* = 68): APPLE, DR-FLASH, FLAME, HATCH, HATCH + OSA, CHADS_2_, R_2_CHADS_2_ CHA_2_Ds_2_VASc, CAAP-AF, PAT_2_C_2_H, C_2_HEST (Supplementary Table S1). Details regarding excluded scores and reasons for exclusion are provided in Supplementary Table S2.

### Atrial cardiac magnetic resonance imaging

Atrial cardiac magnetic resonance imaging was performed on 1.5 Tesla clinical magnetic resonance imaging scanners. Left atrial area assessment was performed in the four-chamber view in ventricular end systole prior to mitral valve opening [11]. Volumetric and functional analysis were performed using the Simpson’s short-axis method as previously described [12]. Following contrast administration, a contrast-enhanced magnetic resonance angiogram (CE-MRA) was obtained to delineate the left atrial endocardial border 90 s after contrast administration. This was followed 20 min later by a three-dimensional late gadolinium enhancement (LGE) image to assess atrial fibrosis burden. Quantitative fibrosis assessment was performed using CEMRGapp (version 1.0, https://www.cemrg.com/) [13]. The signal mean and standard deviation of the left atrial blood pool were used as reference values to define atrial fibrotic tissue. The following reference values were used based on previously published studies: (1) signal intensity 3.3 standard deviations greater than the left atrial blood pool mean [14]; (2) image intensity ratio (IIR) 1.2 x blood pool mean; (3) IIR 1.32 x blood pool mean [15]; and (4) IIR 0.97 x blood pool mean [16]. The surface area of fibrotic tissue was indexed to total left atrial surface area to calculate fibrosis burden. Cardiac magnetic resonance imaging-defined measures of left atrial size, contractile function, fibrosis and left ventricular ejection fraction were used for univariable analysis.

### Electroanatomic mapping data

**Fig. 1:**
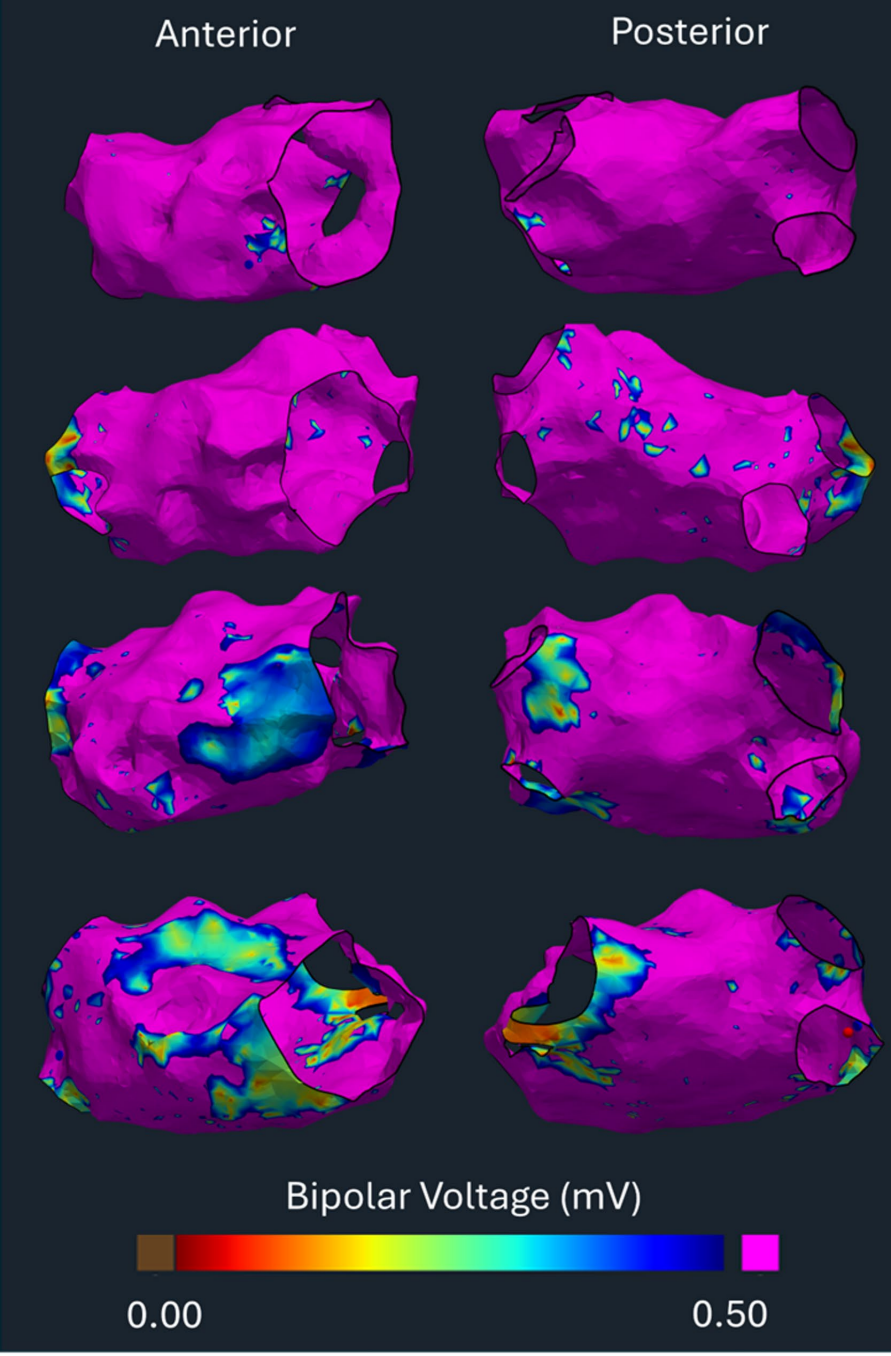
Example left atrial bipolar voltage electroanatomic maps created in EP Workbench, illustrating four different quartiles of mean voltage. The electroanatomic map at the top of the image represents the highest voltage quartile, with decreasing voltage in subsequent maps

Atrial fibrillation ablation procedures were performed under general anaesthesia. A decapolar catheter was placed in the coronary sinus. Following trans-septal puncture, two sheaths and a mapping catheter were advanced into the left atrium. Patients attending in AF underwent external synchronised direct current cardioversion. All electroanatomic maps were created during proximal coronary sinus pacing using Carto3 (Biosense Webster, Diamond Bar) at a constant cycle length of 500 or 600ms (Fig. 1). The pulmonary veins and left atrial appendage were removed using the mapping system tools. Any points greater than 7 mm from the atrial surface were removed.

Quantitative analysis of electroanatomic mapping data was undertaken using OpenEP [17] to calculate mean voltage (mV), percentage of low voltage areas (defined as areas of atrial tissue with voltage < 0.5mV), mean conduction velocity and mean electrogram duration. Voltage was calculated using the interpolated mapping data exported from the clinical mapping system. Conduction velocity was calculated using radial basis interpolation implemented in OpenEP. Electrogram duration was determined by applying the non-linear energy operator [18] to each bipolar electrogram signal within the window of interest. Electrogram durations ± 2 standard deviations from the mean were excluded to reduce the influence of noise and artefacts.

### Outcome measures

Patients were followed up at 3- and 12-months with 24-hour and 12-lead electrocardiogram recordings or more frequently according to clinical status and symptomatology. In time-to-event analysis, AF recurrence was defined as the earliest occurrence of either a documented episode of AF lasting > 30 s or patient-reported recurrence of typical symptoms, confirmed by an electrocardiogram, after a 90-day blanking period.

### Model development

Univariable logistic regression was performed to identify variables associated with arrhythmia recurrence in patients with the full set of parameters necessary for clinical risk score calculation (*n* = 68). Variables with P values ≤ 0.2 from the univariable analysis were included for multivariable analysis. The entire cohort (*n* = 123) was randomly split 70:30 into training and testing datasets, with multivariable analysis performed on the training cohort. Variance inflation factor analysis was used to identify and exclude variables with multicollinearity, where variance inflation factors greater than five indicated significant collinearity [19]. A correlation matrix was also used to examine the correlation between individual predictors.

Four multivariable model types were developed comprising: (1) clinical data only; (2) clinical data and magnetic resonance imaging data; (3) clinical data and electroanatomic mapping data; (4) clinical, magnetic resonance imaging, and electroanatomic mapping data.

### Statistical analysis

Normally distributed continuous variables are presented as mean ± standard deviation. Non-normally distributed or non-continuous data are presented as median (interquartile range). Categorical data are presented as percentages. For continuous variables, t-tests were performed, while Kruskal-Wallis tests were used for ordinal variables. Categorical variables were analysed using Chi-square tests or Fisher’s exact tests depending on the expected frequencies.

Area under the curve receiver operating characteristic (AUC-ROC) analysis was used to compare the ability of clinical risk scores and the combined model to predict arrhythmia recurrence risk after one procedure. The combined model was subjected to 1000 iterations of bootstrap resampling to generate bootstrapped AUC estimates and confidence intervals thereby enabling internal validation [20]. 

Model calibration was assessed using bootstrap resampling to compare predicted probabilities with observed outcomes. The model’s accuracy was quantified using the Brier score [21], which measures the mean squared difference between predicted probabilities and actual outcomes. Decision curve analysis was performed to evaluate the usefulness of the model across a range of risk thresholds by calculating the net benefit of model-based decisions.

For repeat ablation, the ROC optimal cut-off score was used to divide patients into two groups (high and low arrhythmia recurrence risk) for time-to-event analysis. Kaplan-Meier curves were compared using log-rank tests. Statistical analysis was performed using R (version 4.0.2, R Foundation for Statistical Computing, Vienna).

## Results

### Patient characteristics

Pre-procedure cardiac magnetic resonance imaging and electroanatomic mapping data analysis were performed in 123 patients (Table 1). Of these patients, 65 (53%) had persistent AF and 37 (30%) were female. All patients underwent pulmonary vein isolation with additional ablation performed in 53% of patients as previously described [8]. Mean follow-up time was 626 ± 323 days and arrhythmia recurred in 44/123 (36%) of patients with recurrence rates increasing in patients with longer follow-up durations (Supplementary Table S3). Among patients with arrhythmia recurrence, left atrial end diastolic volume was significantly increased while left atrial ejection fraction and mean left atrial voltage were decreased. A total of 38/123 patients underwent repeat ablation procedures (Table 2). Amongst patients undergoing repeat procedures, the mean follow-up time was 403 ± 242 days following the second procedure.

**Table 1:** Baseline patient characteristics

	No recurrence (*n* = 79)	Recurrence (*n* = 44)	*P* value
Clinical data			
Age (years)	59.2 ± 10.8	61.2 ± 10.8	0.337
Female Gender (n (%))	24 (30.4)	13 (29.5)	1.000
Congestive Cardiac failure (n(%))	16 (20.3)	6 (13.6)	0.501
Hypertension (n(%))	24 (30.4)	18 (41.0)	0.326
Diabetes (n(%))	8 (10.1)	2 (4.5)	0.493
Stroke/TIA (n(%))	3 (3.8)	2 (4.5)	0.708
Coronary disease (n(%))	7 (8.9)	8 (18.2)	0.220
Peripheral Vascular Disease (n(%))	2 (2.5)	0 (0)	0.537
CHA_2_DS_2_VASc Median (Q1, Q3)	1 (0.5, 2)	1.5 (1, 2)	0.716
Persistent atrial fibrillation (n (%))	38 (48.1)	27 (61.4)	0.189
eGFR (mL/min/1.73m^2^)	73.9 ± 19.1	74.9 ± 15.7	0.770
Height (cm)	175.3 ± 10.2	174 ± 10.0	0.490
Weight (kg)	89.7 ± 17.2	86.4 ± 18.4	0.322
BMI (kg/m^2^)	29.2 ± 5.2	28.4 ± 5.0	0.390
MRI Parameters			
LVEF (%)	57.7 ± 9.5	56.4 ± 9.8	0.483
LA area (cm^2^)	27.8 ± 6.6	29.1 ± 7.1	0.313
LA End Diastolic Volume (ml)	73.4 ± 34.3	90.3 ± 43.8	0.030
LA End Systolic Volume (ml)	113.0 ± 28.6	123.0 ± 36.3	0.121
LAEF (%)	37.2 ± 16.8	29.5 ± 17.8	0.021
Quantitative fibrosis assessment			
Atrial LGE IIR 3.3 SD above mean	9.6 ± 7.8	12.2 ± 11.2	0.189
Atrial LGE IIR 0.97	91.0 ± 6.2	91.8 ± 6.2	0.486
Atrial LGE IIR 1.22	23.9 ± 19.3	23.8 ± 20.4	0.992
Atrial LGE IIR 1.32	10.1 ± 12.0	9.9 ± 12.4	0.915
Electroanatomic mapping data parameters			
Mean Bipolar Voltage (mV)	2.0 ± 0.6	1.7 ± 0.6	0.002
Mean Conduction Velocity (m/s)	0.8 ± 0.1	0.8 ± 0.1	0.140
Mean Percentage Low Voltage Areas (%)	6.4 ± 8.8	9.7 ± 13.9	0.160
Mean Electrogram Duration (ms)	18.5 ± 2.9	17.8 ± 4.0	0.264

BMI = Body mass index, TIA = Transient Ischaemic attack, eGFR = estimated Glomerular Filtration Rate, LV = left ventricle, EF = ejection fraction, LA = left atrium, LGE = late gadolinium enhancement, IIR = image intensity ratio, SD = standard deviation

**Table 2:** Baseline characteristics of patients undergoing repeat ablation procedures

	Repeat procedure patients (*n* = 38)		*P* value
	No recurrence (*n* = 22)	Recurrence (*n* = 16)	
Clinical data			
Age (years)	61.5 ± 8.0	55.5 ± 14.0	0.141
Female Gender (n (%))	5 (22.7)	6 (37.5)	0.471
Congestive Cardiac failure (n(%))	3 (13.6)	1 (6.3)	0.624
Hypertension (n(%))	9 (40.9)	4 (25.0)	0.490
Diabetes (n(%))	1 (4.5)	1 (6.3)	1.000
Stroke/TIA (n(%))	2 (9.1)	0 (0.0)	0.499
Coronary disease (n(%))	6 (27.3)	2 (12.5)	0.427
Peripheral Vascular Disease (n(%))	0 (0)	0 (0)	N/A
CHA_2_DS_2_VASc Median (Q1, Q3)	2 (1, 2.75)	1 (0, 1.25)	0.103
Persistent atrial fibrillation (n (%))	10 (45.6)	12 (75)	0.100
eGFR (mL/min/1.73m^2^)	77.6 ± 16.1	77.5 ± 21.2	0.983
Height (cm)	175.9 ± 8.4	170.3 ± 10.4	0.083
Weight (kg)	86.2 ± 12.0	82.8 ± 20.5	0.557
BMI (kg/m^2^)	27.8 ± 3.2	28.5 ± 6.5	0.725
MRI Parameters			
LVEF (%)	56.6 ± 10.3	57.4 ± 9.0	0.800
LA area (cm^2^)	28.0 ± 6.3	27.8 ± 7.2	0.923
LA End Diastolic Volume (ml)	82.1 ± 37.4	80.0 ± 32.3	0.853
LA End Systolic Volume (ml)	117.2 ± 29.4	113.6 ± 34.0	0.735
LAEF (%)	32.3 ± 17.5	30.8 ± 14.8	0.774
Quantitative fibrosis assessment			
Atrial LGE IIR 3.3 SD above mean	11.3 ± 9.4	12.6 ± 14.2	0.762
Atrial LGE IIR 0.97	91.9 ± 5.7	92.2 ± 7.2	0.900
Atrial LGE IIR 1.22	23.5 ± 19.6	25.6 ± 25.1	0.789
Atrial LGE IIR 1.32	9.6 ± 11.7	11.7 ± 15.2	0.656
Electroanatomic mapping data parameters			
Mean Bipolar Voltage (mV)	2.0 ± 0.5	1.8 ± 0.5	0.229
Mean Conduction Velocity (m/s)	0.8 ± 0.2	0.8 ± 1.1	0.895
Mean Percentage Low Voltage Areas (%)	6.5 ± 9.8	6.8 ± 7.1	0.898
Mean Electrogram Duration (ms)	18.5 ± 3.2	18.4 ± 2.9	0.938

BMI = Body mass index, TIA = Transient Ischaemic attack, eGFR = estimated Glomerular Filtration Rate, LV = left ventricle, EF = ejection fraction, LA = left atrium, LGE = late gadolinium enhancement, IIR = image intensity ratio, SD = standard deviation

### Predictors of clinical recurrence

In the univariable analysis, age, weight, hypertension, left atrial end diastolic volume, left atrial end systolic volume, left atrial ejection fraction, and mean left atrial voltage had P values ≤ 0.2 (Table 3). Initial variance inflation factor analysis revealed collinearity between left atrial ejection fraction, left atrial end diastolic volume and end systolic volume (variance inflation factors > 5). This was further supported by correlation analysis which demonstrated inverse correlations between left atrial ejection fraction, and both left atrial end diastolic volume (*r*= -0.86) and end systolic volume (*r*= -0.55) (Supplementary Figure S1). Left atrial end diastolic volume and end systolic volume were subsequently removed from the model. Variance inflation factors for the remaining variables were all less than 5 (age 1.21, weight 1.12, hypertension 1.21, left atrial ejection fraction 1.34, mean voltage 1.66) and subsequently included in the multivariable analysis.

**Table 3:** Univariable and multivariable analysis results

Variables	Univariable analysis			Multivariable analysis		
Characteristic	Odds Ratio	95% Confidence interval	*P* value	Odds Ratio	95% Confidence interval	*P* value
Age	1.05	0.99, 1.12	0.13	0.97	0.92, 1.02	0.21
Gender	0.98	0.32, 2.89	> 0.9			
Congestive cardiac failure	0.90	0.22, 3.24	0.9			
Hypertension	2.02	0.72, 5.73	0.2	1.82	0.66, 5.19	0.25
Diabetes	0.41	0.06, 1.82	0.3			
Stroke/TIA	0.63	0.05, 2.69	0.6			
Coronary artery disease	2.05	0.51, 8.24	0.3			
Peripheral Vascular Disease	0.00		> 0.9			
Persistent atrial fibrillation	1.53	0.57, 4.27	0.4			
eGFR (mL/min/1.73m^2^)	1.01	0.98, 1.04	0.4			
Height (cm)	0.99	0.93, 1.04	0.6			
Weight (kg)	0.98	0.95, 1.01	0.2	0.99	0.96, 1.02	0.53
BMI (kg/m^2^)	0.95	0.85, 1.05	0.3			
MRI Parameters						
LVEF (%)	0.99	0.94, 1.04	0.7			
LA area (cm^2^)	1.04	0.97, 1.12	0.3			
LA End Diastolic Volume (ml)	1.02	1.00, 1.03	0.041			
LA End Systolic Volume (ml)	1.02	1.00, 1.04	0.087			
LAEF (%)	0.97	0.94, 1.00	0.042	0.99	0.95, 1.01	0.34
Quantitative fibrosis assessment						
Atrial LGE 3.3 SD above mean	1.03	0.97, 1.10	0.4			
Atrial LGE IIR 0.97	1.01	0.92, 1.10	0.9			
Atrial LGE IIR 1.22	1.01	0.98, 1.03	0.7			
Atrial LGE IIR.32	1.02	0.97, 1.07	0.5			
Electroanatomic mapping data parameters						
Mean Voltage (mV)	0.44	0.16, 1.06	0.081	0.42	0.14, 1.13	0.10
Percentage of Low Voltage Areas (%)	1.03	0.95, 1.11	0.5			
Mean Conduction Velocity (m/s)	0.12	0.00, 9.70	0.4			
Mean Electrogram Duration	1.00	0.84, 1.21	> 0.9			

BMI = Body mass index, TIA = Transient Ischaemic attack, eGFR = estimated Glomerular Filtration Rate, LV = left ventricle, EF = ejection fraction, LA = left atrium, LGE = late gadolinium enhancement, IIR = image intensity ratio, SD = standard deviation

### Predicting response to index procedures

**Fig. 2:**
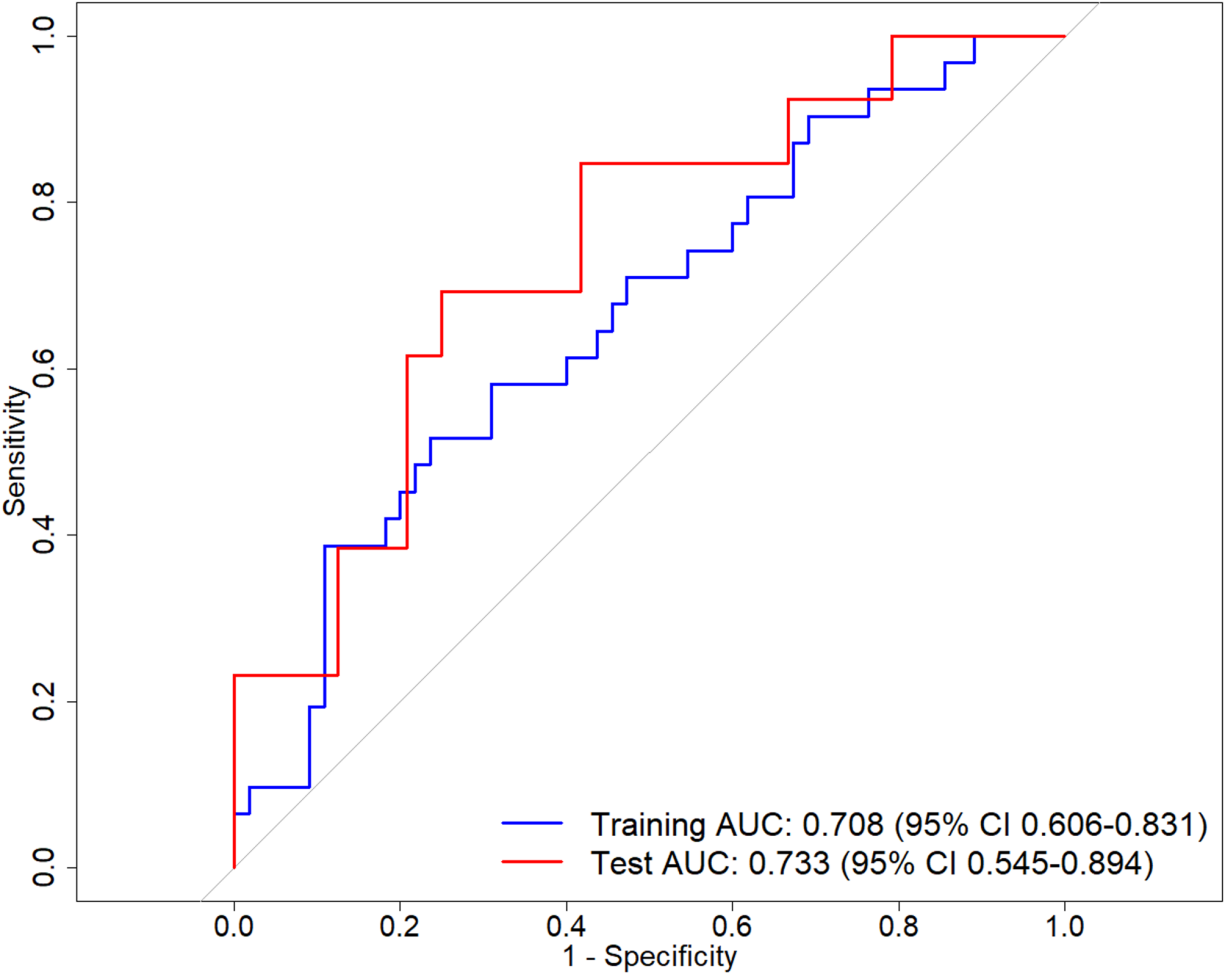
Area under the curve-receiver operating characteristic analysis for the developed multivariable logistic regression model (comprising age, weight, hypertension, left atrial ejection fraction and mean left atrial voltage) for predicting atrial fibrillation/tachycardia recurrence at 12 months

The bootstrapped multivariable logistic regression model achieved an AUC of 0.708 (95% confidence interval (CI) 0.606–0.813) in the training cohort and 0.733 (95% CI 0.545–0.894) in the testing cohort for predicting arrhythmia recurrence at 12 months (Fig. 2). Calibration assessed via 1000 bootstrap resamples demonstrated partial agreement between predicted and observed probabilities (Supplementary Figure S2) with Brier scores of 0.211 in the training dataset and 0.197 in the testing dataset. Decision curve analysis demonstrated that the net benefit curve of the model was higher than “all” and “none” at most threshold probabilities (Supplementary Figure S3).

**Fig. 3:**
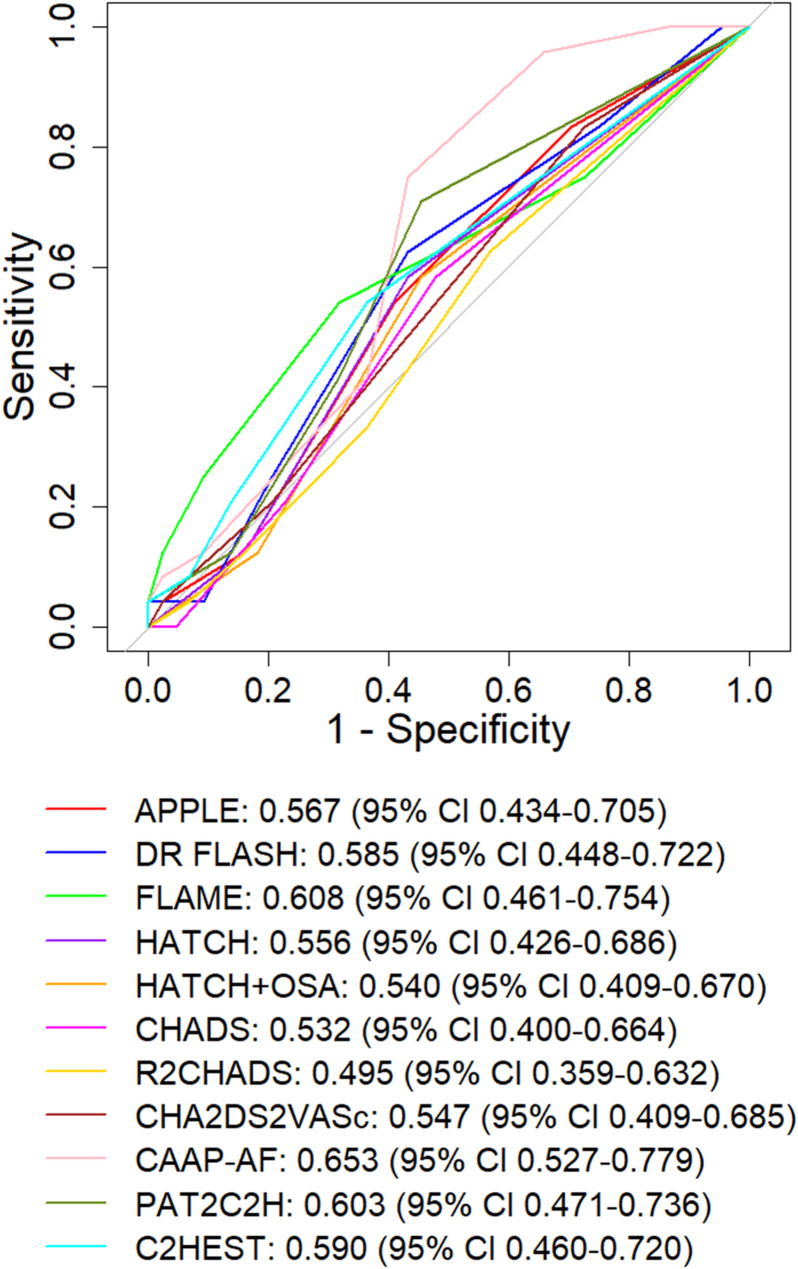
Area under the curve-receiver operating characteristic analysis for currently available clinical risk predictive models for predicting atrial fibrillation/tachycardia recurrence at 12 months

The combined model outperformed other multivariable logistic regression models comprising (1) clinical data only, (2) clinical and magnetic resonance imaging data; and (3) clinical and electroanatomic mapping data (Table 4). The final trained logistic regression model is available for research use as a Work-in-Progress module for EP Workbench [17, 22] (Supplementary Figure S4, https://github.com/ep-workbench-wips/Bodagh-2025-AF-MRI-EAM-Inference-Model). Amongst clinical risk prediction tools, CAAP-AF had the highest predictive value (AUC 0.653, 95% CI 0.527–0.779) (Fig. 3). Only two other clinical scores (FLAME and PAT_2_C_2_H) achieved an AUC of 0.6 or greater.

**Table 4:** Bootstrapped multivariable logistic regression model AUCs for different combinations of data across training, testing, and repeat procedure datasets

Model Type	Dataset		
	Training	Test	Repeat procedure
Combined model	0.708 (95% CI 0.606–0.813)	0.733 (95% CI 0.545–0.894)	0.680 (95% CI 0.509–0.852)
Clinical data only	0.619 (95% CI 0.453–0.728)	0.610 (95% CI 0.415–0.805)	0.564 (95% CI 0.436–0.719)
Clinical + magnetic resonance imaging data	0.678 (95% CI 0.570–0.786)	0.677 (95% CI 0.570–0.786)	0.586 (95% CI 0.450–0.750)
Clinical + electroanatomic mapping data	0.686 (95% CI 0.575–0.784)	0.700 (95% CI 0.462–0.873)	0.662 (95% CI 0.409–0.849)

clinical data: age, weight, hypertension; magnetic resonance imaging data = left atrial ejection fraction; electroanatomic mapping data: mean voltage

### Predicting response to repeat procedures

**Fig. 4:**
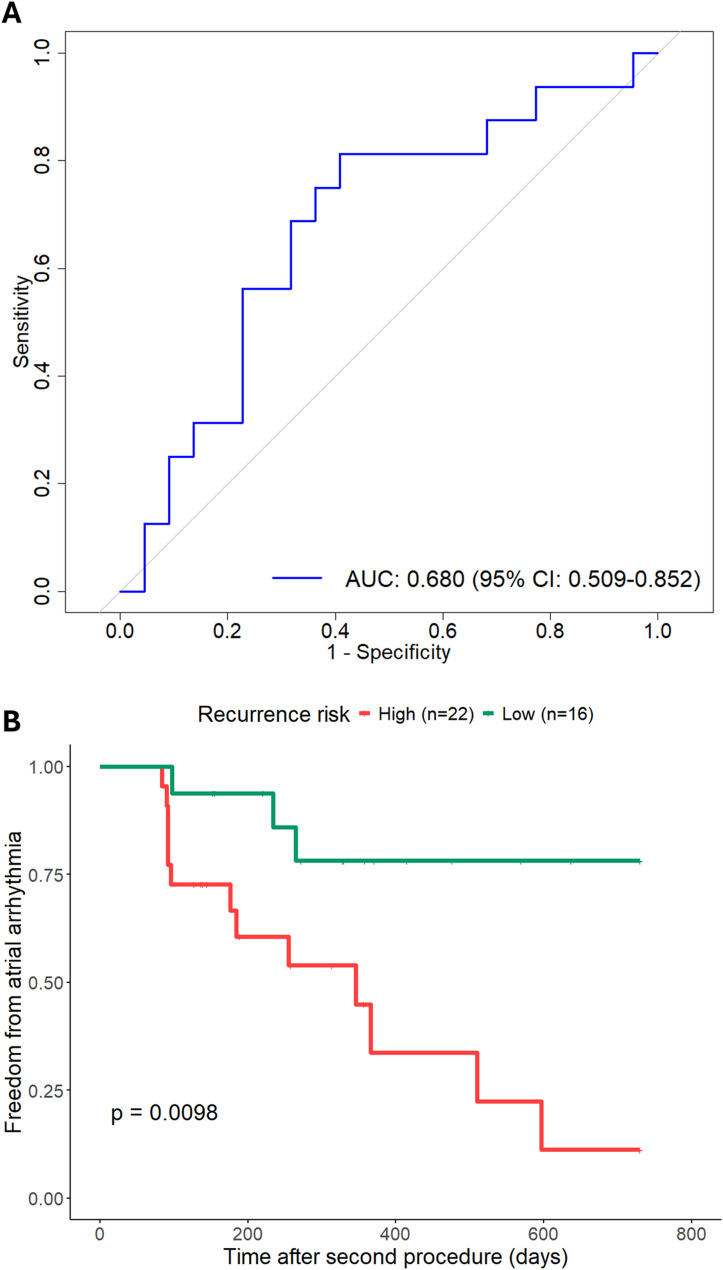
(**A**) Area under the curve-receiver operating characteristic (AUC-ROC) analysis for the multivariable logistic regression model (comprising age, weight, hypertension, left atrial ejection fraction and mean left atrial voltage) in predicting atrial fibrillation/tachycardia recurrence following repeat ablation. (**B**) Kaplan-Meier curves for atrial fibrillation recurrence. The ROC optimal cut-off score was used to divide patients into two groups (high recurrence risk, low recurrence risk) for time-to-event analysis

In patients undergoing repeat ablation procedures, the combined model achieved an AUC of 0.680 (95% CI 0.509–0.852) for predicting arrhythmia recurrence (Fig. 4A), with high precision (0.813) and specificity (0.813) (Supplementary Table S4). The combined model, which incorporated clinical, magnetic resonance imaging, and electroanatomic mapping data, demonstrated superior performance compared to other models that lacked one or more of these data modalities (Table 4). The optimal cutoff for predicting recurrence risk was determined using the ROC curve to maximise Youden’s index. Kaplan-Meier curves showed a higher risk of arrhythmia recurrence following repeat ablation in the high recurrence risk group (log-rank *p* = 0.010) (Fig. 4B).

## Discussion

This study investigated whether a predictive model incorporating left atrial electromechanical remodelling data could improve the prediction of outcomes following AF catheter ablation. The principal findings were that (1) a multivariable logistic regression model comprising clinical, pre-procedure magnetic resonance imaging and index-procedure electroanatomic mapping data outperformed eleven published risk scores in predicting index procedure response in our patient cohort; (2) the developed model may improve the prediction of treatment response for repeat procedures. These findings demonstrate the potential for improved treatment response prediction using measurement of the extent of atrial electromechanical remodelling and, if confirmed in larger studies, highlight the opportunity to inform patient decision-making for repeat procedures.

### Current clinical risk prediction tools

Several clinical risk scores have been developed to predict arrhythmia recurrence risk following AF ablation, but subsequent studies have shown limited performance in external patient cohorts [5, 6]. Our results affirm these findings with the highest performing clinical risk score (CAAP-AF) attaining an AUC 0.653 (95% CI 0.527–0.779). The limited performance of clinical scores in external cohorts is likely to reflect the composition of these scores which typically comprise a combination of clinical parameters and an echocardiographic parameter describing either left atrial size or left ventricular function. As such, they do not directly delineate the extent of electromechanical remodelling present.

Other studies have attempted to correlate electrocardiogram-derived P-wave duration with AF recurrence risk following ablation [23]. Whilst this provides some insight into atrial conduction, it remains limited in its ability to quantify the degree of atrial electromechanical remodelling. Owing to the contemporary availability of both magnetic resonance imaging and electrophysiological data which directly quantify atrial remodelling, we therefore sought to develop a novel method to predict AF catheter ablation outcomes using direct measurements of atrial anatomical and functional properties.

### Identifying electromechanical changes to predict recurrence

We postulated that magnetic resonance imaging and electroanatomic mapping data analysis could identify electromechanical changes conducive to arrhythmia recurrence, and that their measurement would inform an improved prediction model for arrhythmia recurrence. Univariable analysis identified pre-procedural magnetic resonance imaging derived left atrial ejection fraction as a risk factor for recurrence following ablation, which is consistent with previous studies demonstrating an association between magnetic resonance imaging derived measures of left atrial function and arrhythmia recurrence [24]. 

In addition, univariable analysis revealed mean left atrial voltage as a predictor of AF recurrence, which is also consistent with previous studies showing a correlation between voltage and AF recurrence post-ablation [25, 26]. Areas of low voltage have been associated with atrial fibrosis, increased intercellular space, myofibrillar loss and decreased myocardial nuclear density [27]. Voltage mapping is likely to identify a combination of structural atrial changes, many of which may contribute to arrhythmia recurrence.

Late gadolinium enhancement magnetic resonance imaging has also been employed to identify structural atrial changes associated with arrhythmia recurrence. However, quantitative magnetic resonance imaging fibrosis assessment was not identified as a significant predictor of outcomes following AF catheter ablation in our analysis. This could potentially reflect limitations in the ability of magnetic resonance imaging to accurately characterise the atrial substrate using existing analysis techniques, underscoring the utility of electroanatomic voltage mapping as a more direct method for arrhythmogenic substrate assessment.

### Predicting repeat procedure response from index procedure data

Following pulmonary vein isolation, recurrence of AF is associated with pulmonary vein to left atrium reconnection and/or the presence of pro-arrhythmic triggers/substrate which are not confined to the pulmonary vein antra [28]. Durable pulmonary vein isolation is achieved in approximately 71–87% of pulmonary veins depending on the energy source used [29]. Beyond the pulmonary veins, routinely acquired electroanatomic mapping can identify perturbations in the electrical substrate that may be important for the initiation and maintenance of arrhythmia, and therefore for recurrence despite durable index pulmonary vein isolation.

Our findings demonstrate how the identification of such perturbations from the index procedure can be utilised to develop a predictive model capable of optimising patient selection for repeat procedures. In this study, electroanatomic mapping data were acquired using a uniform pacing protocol, thereby facilitating a standardised approach. Future studies should examine whether our findings extend to cases where maps are collected during sinus rhythm or AF as this may affect the measurement of parameters such as voltage, conduction velocity and electrogram duration.

### Incorporating additional imaging modalities to improve outcome prediction

This study investigated how two modalities (magnetic resonance imaging and electroanatomic mapping) can be combined to delineate the extent of atrial electromechanical remodelling to improve AF ablation outcome prediction. Future research could investigate whether incorporation of, or replacement with, additional imaging modalities could further improve predictive accuracy. For example, computed tomography has excellent spatial resolution and enables precise assessment of left atrial and pulmonary venous anatomy [30]. This modality has been used to demonstrate that structural parameters—such as left atrial wall thickness, sphericity, volume, ejection fraction, and pulmonary vein morphology—are associated with arrhythmia recurrence following catheter ablation [31]. Computed tomography has also emerged as a potential tool for the assessment of left atrial fibrosis [32], and may therefore facilitate the identification of atrial structural and mechanical remodelling.

Speckle tracking echocardiography is another non-invasive technique capable of facilitating atrial strain analysis, and numerous studies have shown reduced left atrial strain to be a predictor of AF recurrence following catheter ablation [31]. Additional echocardiographic techniques, including electromechanical cycle length mapping [33, 34], and total atrial conduction time [35] have also been developed to non-invasively quantify the degree of atrial electromechanical remodelling and been shown to be predictive of AF ablation outcomes.

Echocardiographic strain imaging and computed tomography may therefore enable improved identification of atrial electromechanical changes conducive to arrhythmogenesis, and the incorporation of these or other modalities in future work may further improve predictive accuracy and clinical utility. Future studies should determine the most effective methods for quantifying imaging-derived parameters to accurately reflect the spectrum of atrial electromechanical changes that facilitate arrhythmogenesis.

### Clinical relevance

A model to predict response to repeat procedures has the potential to enhance the care pathway for AF patients. Arrhythmia recurrences are seen following a repeat ablation in approximately 25–45% of patients [3, 4], and repeat procedures incur a 1–2% risk of major complications [36]. An improved patient selection process focused on identifying those patients in whom the likelihood of treatment success is highest could improve clinical outcomes, reduce the burden of complications and offer financial benefits for healthcare systems globally.

### Limitations

Our study has shown how the extent of atrial electromechanical remodelling can be utilised to improve AF ablation outcome prediction. However, the analysis was based on a single-centre cohort (*n* = 123), with 38 patients undergoing repeat ablation procedures. The study is therefore underpowered to estimate the odds ratio associated with each model parameter, as reflected by the wide 95% confidence intervals. This limited power may also explain why certain trends—such as the higher proportion of patients with persistent AF in the recurrence group—did not reach statistical significance. Based on the approach outlined by Hajian-Tilaki [37], we calculate that a sample size of at least 534 patients would be required to develop a model with diagnostic accuracy AUC > 0.70, marginal error ± 5% and a 95% confidence interval, based on an assumed repeat AF ablation recurrence rate of 43.4% [38]. Prior studies have shown that current predictive models have an AUC of approximately 0.60. A model achieving an AUC > 0.70 would therefore represent a significant improvement in ablation outcome prediction beyond existing approaches.

An additional limitation of our study is the variability of the ablation strategies employed. Larger, multicentre studies with external validation datasets are therefore needed to account for procedural heterogeneity, mitigate the risk of centre-specific bias and test model generalisation. Such studies could confer improvements in calibration and net clinical benefit to further strengthen the clinical utility of predictive models. The findings presented in this study should be considered indicative of the potential for combined assessment to enhance outcome prediction, but further model development and external validation are required to confirm their clinical applicability. Variability in follow-up duration may also have influenced the observed rates of arrhythmia recurrence. Whilst our study benefitted from a lengthy average follow-up, detection bias may have influenced recurrence estimates and model performance.

## Conclusion

Outcome prediction following AF catheter ablation may be enhanced through the integration of multimodal data delineating the extent of atrial electromechanical remodelling into predictive models. The application of predictive models incorporating patient-specific atrial electromechanical data in clinical workflows could optimise patient selection for repeat AF ablation procedures.

## Supplementary Information

Below is the link to the electronic supplementary material.

Supplementary Material 1

## Data Availability

The dataset generated and analysed during the current study is not publicly available due to the presence of potentially sensitive personal information. However, de-identified data may be made available from the corresponding author on reasonable request, subject to approval by a relevant institutional review board or ethics committee. The final trained logistic regression model is available for research use as a Work-in-Progress module for EP Workbench (Supplementary Figure S4, https://github.com/ep-workbench-wips/Bodagh-2025-AF-MRI-EAM-Inference-Model).

## Abbreviation

AF: Atrial fibrillation
