# Supplementary material for: Combining clinical and left atrial electromechanical remodelling data: potential to improve atrial fibrillation ablation outcome prediction
Source: BMC Med Inform Decis Mak. 2025 Sep 29;25:356. doi: 10.1186/s12911-025-03200-7 (PMC12482448; doi:10.1186/s12911-025-03200-7)
Supplement: Supplementary file 1 — Supplementary Material 1 [file 12911_2025_3200_MOESM1_ESM.docx]

# Supplementary material

**Supplementary table S1.** Composition of clinical risk scores calculated in patients with the full set of parameters necessary for risk score calculation (n=68). AF = atrial fibrillation, AAD = antiarrhythmic drug, TIA = transient ischaemic attack, COPD = chronic obstructive pulmonary disease.

| Variables | APPLE^1^ | DR-FLASH ^2^ | FLAME^3^ | HATCH^4^ | HATCH+OSA^5^ | CHADS_2_^6^ | R_2_CHADS_2_^7^ | CHA_2_Ds_2_VASc^8^ | CAAP-AF^9^ | PAT_2_C_2_H ^10^ | C_2_HEST^11^ |
| --- | --- | --- | --- | --- | --- | --- | --- | --- | --- | --- | --- |
| Age | X |  |  | X | X | X | X | X | X | X | X |
| Sex |  | X | X |  |  |  |  | X |  |  |  |
| Type of AF | X | X |  |  |  |  |  |  | X | X |  |
| Duration of AF |  |  | X |  |  |  |  |  |  |  |  |
| AADs failed |  |  |  |  |  |  |  |  | X |  |  |
| Heart failure |  |  |  | X | X | X | X | X |  | X | X |
| Diabetes mellitus |  | X |  |  |  | X | X | X |  |  |  |
| Hypertension |  | X |  | X | X | X | X | X |  | X | X |
| TIA/Stroke |  |  |  | X | X | X | X | X |  | X |  |
| COPD |  |  |  | X | X |  |  |  |  | X | X |
| Mitral regurgitation |  |  | X |  |  |  |  |  |  |  |  |
| Obstructive Sleep Apnoea |  |  |  |  | X |  |  |  |  |  |  |
| Coronary artery disease |  |  |  |  |  |  |  |  | X |  | X |
| Vascular disease |  |  |  |  |  |  |  | X |  |  |  |
| Thyroid disease |  |  |  |  |  |  |  |  |  |  | X |
| Extreme comorbidity* |  |  | X |  |  |  |  |  |  |  |  |
| eGFR | X | X |  |  |  |  | X |  |  |  |  |
| Echocardiogram-derived Left atrial diameter | X | X | X |  |  |  |  |  | X | X |  |
| Echocardiogram-derived Left ventricular ejection fraction | X |  |  |  |  |  |  |  |  |  |  |

*Extreme comorbidity is defined as one of the following: severe mitral regurgitation, moderate or greater mitral stenosis, mitral valve replacement, hypertrophic cardiomyopathy, structural congenital heart disease.

**Supplementary Table S2.** Additional clinical risk scores considered but not included. AF = Atrial fibrillation, LA = left atrium.

| Score | Components | Reason for exclusion |
| --- | --- | --- |
| ALARMEc^12^ | AF type (A), Left Atrial size [normalized left atrial area (NLA) ≥10.25], Renal insufficiency (eGRF <68 ml/min), Metabolic syndrome and cardiomyopathy. | Left atrial area measurements unavailable for 61 patients |
| ATLAS^13^ | A, Age; T, Type of AF; LA, LA volume; S, Sex and Smoking | Left atrial volume data unavailable in 108 patients |
| BASE-AF2^14^ | Body mass index >28 kg/m^2^(2) (1); Atrial dilatation >40 mm (1); current Smoking (1); Early recurrence (1); duration of AF history >6 years (1) and non-paroxysmal type (1) of AF | Smoking data unavailable in 113 patients. |
| BNP + (1) CHADS_2_; (2) CHA_2_DS_2_-VASc, (3) R_2_CHADS_2_, and (4) HATCH.  All risk scores derived from ^15^ | CHADS_2_ + BNP  CHA_2_DS_2_-VASc + BNP  R_2_CHADS_2_ + BNP  HATCH + BNP | BNP data unavailable in almost all patients |
| FER2CI^16^ | Female, early recurrence of atrial fibrillation (within 3 months), shorter minimum coupling time (CI) of atrial premature contraction (< 48%) calculated from 24-hour Holter recording at 12 months. | Predicts late recurrence of atrial fibrillation. |
| MB-LATER^17^ | Male, Bundle brunch block, Left atrium ≥47 mm, Type of AF [paroxysmal, persistent or long-standing persistent], and ER-AF = early recurrent AF | Predicts late atrial fibrillation recurrence. |
| Novel risk model comprising age, coronary artery disease (CAD), heart failure (HF), hypertension, transient ischemic attack (TIA) or cerebrovascular accident (CVA), and left atrial diameter (LAD)^18^ | Age, coronary artery disease (CAD), heart failure (HF), hypertension, transient ischemic attack (TIA) or cerebrovascular accident (CVA), and left atrial diameter (LAD) | Hypertension grades not known in the patient cohort. |
| PRE_2_SSS_2_ score^19^ | [PRE]vious ablation: 2 points, female [S]ex: 1 point, [S]inus node dysfunction: 1 point, left atrial [S]car: 2 points | The patients included in this study underwent first time atrial fibrillation ablation procedures. |
| SUCCESS^20^ | Severity of AF type (S), Unsuccessful previous ablations (U), Creatinine Clearance <60 mL/min (CC), Elderly (E), Size of LA (S), Systolic left ventricular EF (S). | The SUCCESS score is comprised of the APPLE score with an additional point for patients undergoing repeat ablation procedures. The patients studied in this study underwent first time atrial fibrillation ablation. |
| VAT-DHF Score^21^ | [V]olume: 1 point, [A]F [T]ype: 1 point, [D]iabetes: 1 point, [H]eight: 1 point, [F]-waves: 1 point | Left atrial volume data unavailable in 108 patients. |

**Supplementary Table S3.** Arrhythmia recurrence rates in patients stratified into quartiles according to the follow up duration after (A) index and (B) repeat ablation.

| 1. Index ablation | | | | | |
| --- | --- | --- | --- | --- | --- |
| Quartile | Number of patients | Number of arrhythmia recurrences | Recurrence rate (%) | Median follow up (Days) | Follow up range (Days) |
| Q1 | 31 | 6 | 19.4 | 297 | 112-371 |
| Q2 | 30 | 9 | 30 | 461.5 | 372-568 |
| Q3 | 31 | 15 | 48.4 | 657 | 570-810 |
| Q4 | 31 | 14 | 45.2 | 1073 | 818-1629 |
| 1. Repeat ablation | | | | | |
| Quartile | Number of patients | Number of arrhythmia recurrences | Recurrence rate (%) | Median follow up (Days) | Follow up range (Days) |
| Q1 | 10 | 3 | 30 | 142 | 14-214 |
| Q2 | 9 | 1 | 11.1 | 314 | 220-358 |
| Q3 | 9 | 6 | 66.7 | 420 | 366-510 |
| Q4 | 10 | 6 | 60 | 727.5 | 569-986 |

**Supplementary Table S4.** Accuracy, precision, recall, specificity and F1 score of the multivariable logistic regression model in the training and test cohorts, as well as among patients undergoing repeat atrial fibrillation ablation procedures

|  | Accuracy | Precision | Recall | Specificity | F1 Score |
| --- | --- | --- | --- | --- | --- |
| Training | 0.674 | 0.737 | 0.764 | 0.516 | 0.750 |
| Test | 0.676 | 0.700 | 0.875 | 0.308 | 0.778 |
| Repeat | 0.684 | 0.813 | 0.591 | 0.813 | 0.684 |

**Supplementary Figure S1.** Correlation matrix of variables with p values ≤0.2 in the initial univariable analysis. LA = Left atrium.


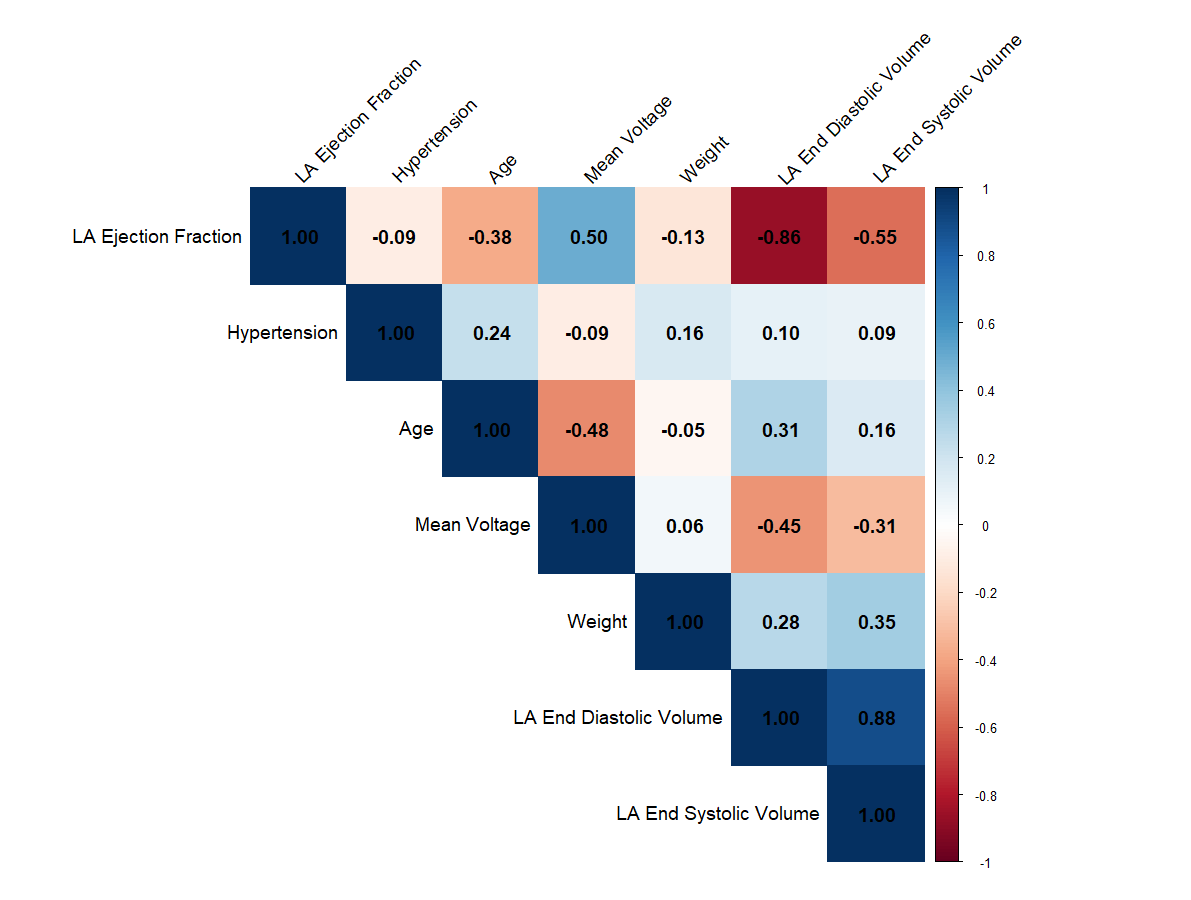


**Supplementary Figure S2.** Calibration plot of the logistic regression model in (A) training and (B) testing datasets.


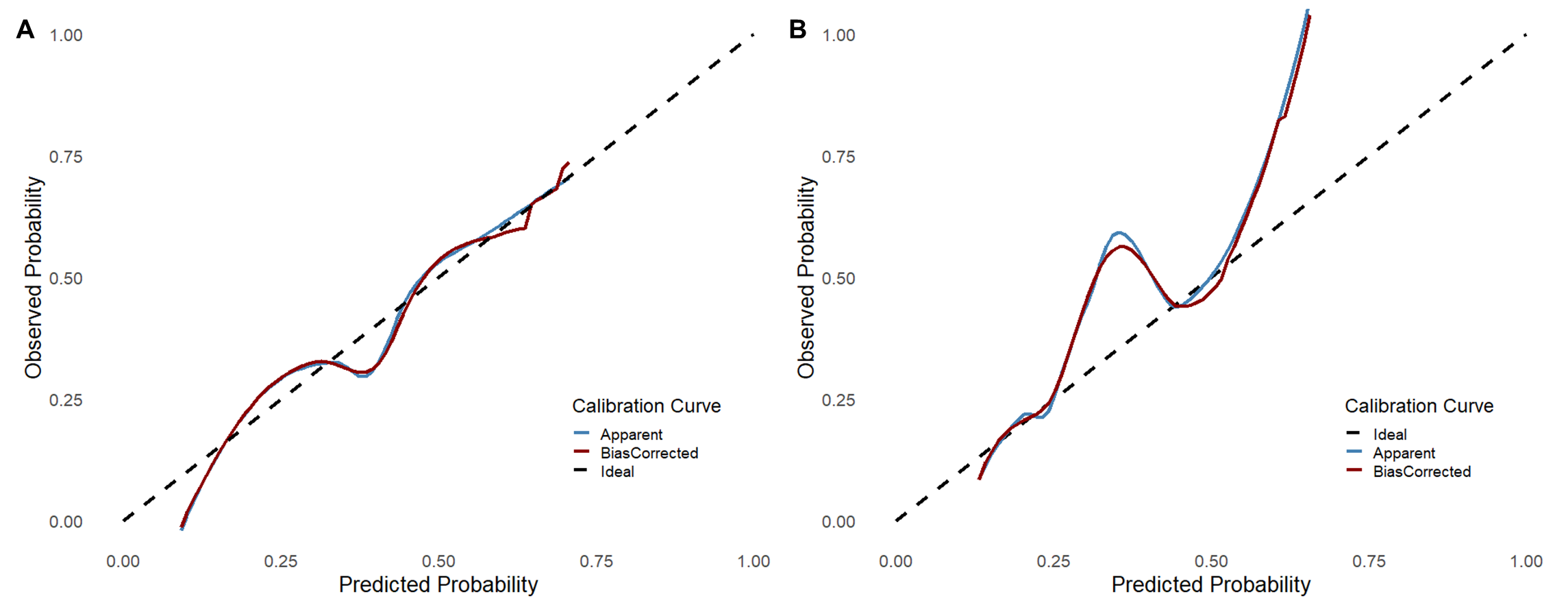


**Supplementary Figure S3.** Decision curve analysis for the prediction model. The grey line represents the strategy where all patients are treated, and the solid black horizontal line represents the strategy where no patients are treated.


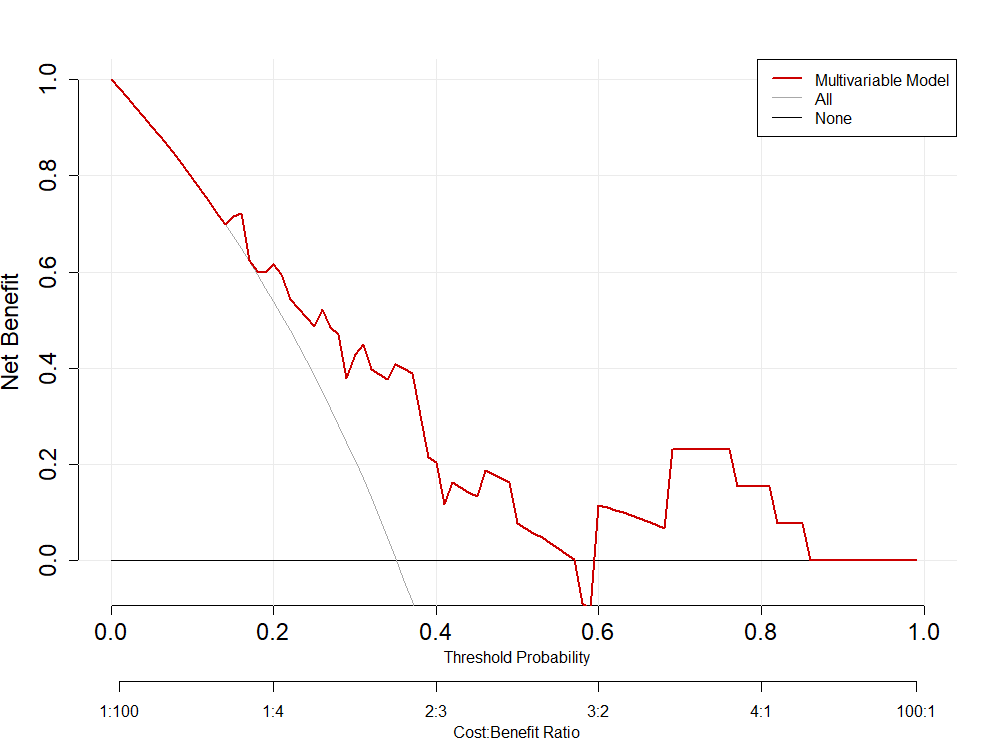


**Supplementary Figure S4.** Screenshot of the trained logistic regression model in EP Workbench ^22,23^. Available as a Work-in-Progress module at <https://github.com/ep-workbench-wips/Bodagh-2025-AF-MRI-EAM-Inference-Model>. The left panel shows a left atrial voltage map. The middle panel shows the Work-In-Progress Workspace containing the specified case object (case_1: Left Atrium) and “Custom Widgets” (variables included in the multivariable model). The Work-In-Progress module is run by pressing the arrow next to the “Code” icon (top right). A pop-up appears the displaying the output, which presents the log odds ratio.


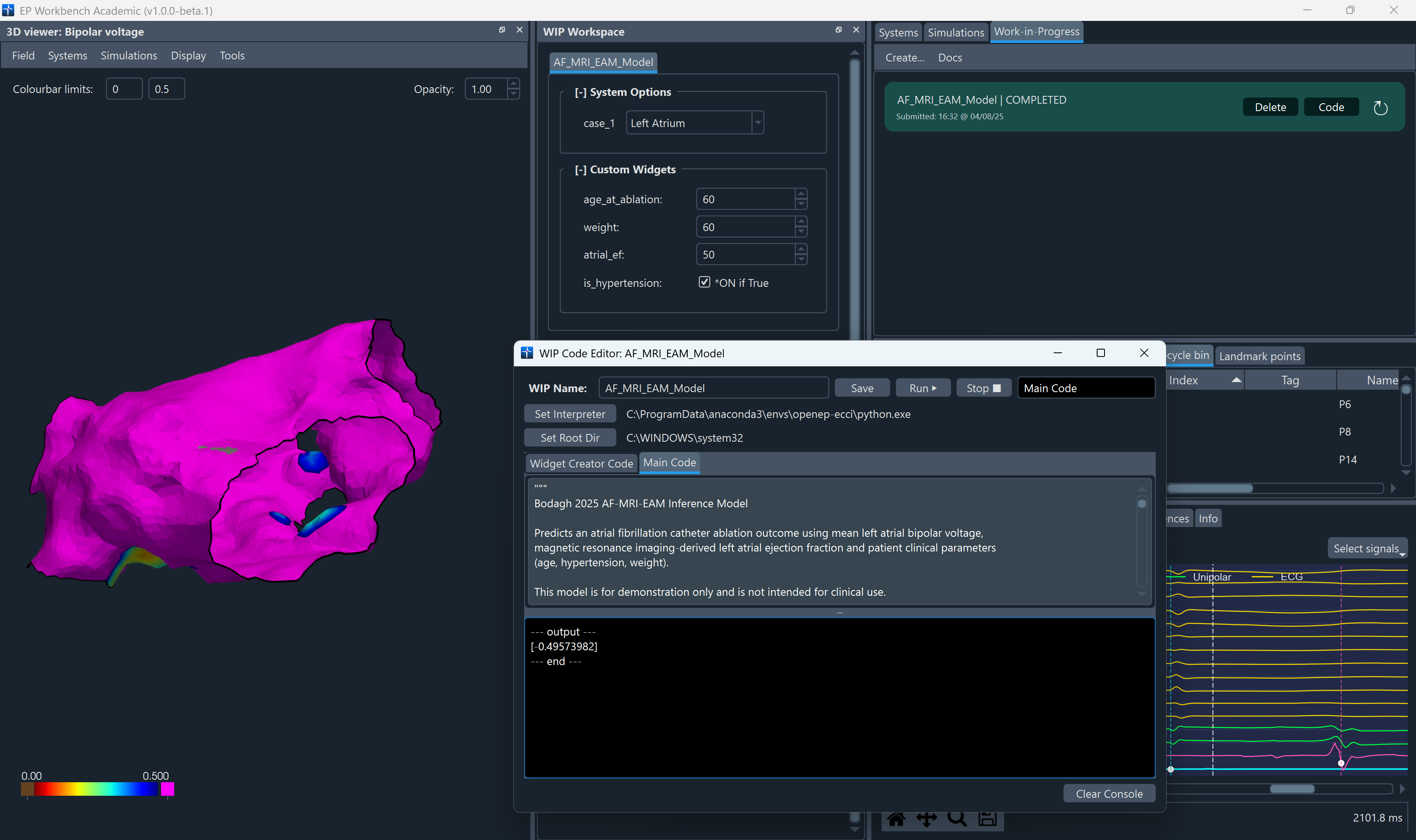


# References

1. Kornej J, Hindricks G, Arya A, et al. The APPLE Score – A Novel Score for the Prediction of Rhythm Outcomes after Repeat Catheter Ablation of Atrial Fibrillation. *PLoS One*; 12. Epub ahead of print 1 January 2017. DOI: 10.1371/JOURNAL.PONE.0169933.

2. Kosiuk J, Dinov B, Kornej J, et al. Prospective, multicenter validation of a clinical risk score for left atrial arrhythmogenic substrate based on voltage analysis: DR-FLASH score. *Heart Rhythm* 2015; 12: 2207–2212.

3. Boyalla V, Jarman JWE, Markides V, et al. Internationally validated score to predict the outcome of non-paroxysmal atrial fibrillation ablation: the ‘FLAME score’. *Open Heart*; 8. Epub ahead of print 4 August 2021. DOI: 10.1136/OPENHRT-2021-001653.

4. De Vos CB, Pisters R, Nieuwlaat R, et al. Progression From Paroxysmal to Persistent Atrial Fibrillation Clinical Correlates and Prognosis. Epub ahead of print 2010. DOI: 10.1016/j.jacc.2009.11.040.

5. Shaikh AY. Modified Hatch Score Predicts 6-Month Recurrence of Atrial Fibrillation after Pulmonary Vein Isolation: Data from the University Of Massachusetts Atrial Fibrillation Registry, https://escholarship.umassmed.edu/cts_retreat (accessed 22 May 2024).

6. Jacobs V, May HT, Bair TL, et al. The impact of risk score (CHADS2 versus CHA2DS2-VASc) on long-term outcomes after atrial fibrillation ablation. *Heart Rhythm* 2015; 12: 681–686.

7. Kornej J, Hindricks G, Kosiuk J, et al. Comparison of CHADS2, R2CHADS2, and CHA2DS2-VASc Scores for the Prediction of Rhythm Outcomes After Catheter Ablation of Atrial Fibrillation. *Circ Arrhythm Electrophysiol* 2014; 7: 281–287.

8. Chao TF, Lin YJ, Tsao HM, et al. CHADS 2 and CHA 2DS 2-VASc scores in the prediction of clinical outcomes in patients with atrial fibrillation after catheter ablation. *J Am Coll Cardiol* 2011; 58: 2380–2385.

9. Winkle RA, Jarman JWE, Mead RH, et al. Predicting atrial fibrillation ablation outcome: The CAAP-AF score. *Heart Rhythm* 2016; 13: 2119–2125.

10. Cay S, Kara M, Ozcan F, et al. A new scoring system: PAT2C2H score. Its clinical use and comparison with HATCH and CHA2DS2-VASc scores in predicting arrhythmia recurrence after cryoballoon ablation of paroxysmal atrial fibrillation. *J Interv Card Electrophysiol* 2022; 65: 701–710.

11. Levent F, Kanat S, Tutuncu A. Predictive Value of C2HEST Score for Atrial Fibrillation Recurrence Following Successful Cryoballoon Pulmonary Vein Isolation in Paroxysmal Atrial Fibrillation. *Angiology* 2023; 74: 273–281.

12. Wójcik M, Berkowitsch A, Greiss H, et al. Repeated catheter ablation of atrial fibrillation: how to predict outcome? *Circ J* 2013; 77: 2271–2279.

13. Mesquita J, Ferreira AM, Cavaco D, et al. Development and validation of a risk score for predicting atrial fibrillation recurrence after a first catheter ablation procedure - ATLAS score. *Europace* 2018; 20: f428–f435.

14. Canpolat U, Aytemir K, Yorgun H, et al. A proposal for a new scoring system in the prediction of catheter ablation outcomes: promising results from the Turkish Cryoablation Registry. *Int J Cardiol* 2013; 169: 201–206.

15. Shaikh AY, Esa N, Martin-Doyle W, et al. Addition of B-type Natriuretic Peptide to Existing Clinical Risk Scores Enhances Identification of Patients at Risk for Atrial Fibrillation Recurrence after Pulmonary Vein Isolation. DOI: 10.1097/HPC.0000000000000060.

16. Abstract 12589: Novel Score to Predict Very Late Recurrence of Atrial Fibrillation After Successful Atrial Fibrillation Catheter Ablation | Circulation, https://www.ahajournals.org/doi/10.1161/circ.140.suppl_1.12589 (accessed 21 May 2024).

17. Mujović N, Marinković M, Marković N, et al. Prediction of very late arrhythmia recurrence after radiofrequency catheter ablation of atrial fibrillation: The MB-LATER clinical score OPEN. *Nature Publishing Group*. Epub ahead of print 2016. DOI: 10.1038/srep40828.

18. Li G, Wang X, Han JJ, et al. Development and validation of a novel risk model for predicting atrial fibrillation recurrence risk among paroxysmal atrial fibrillation patients after the first catheter ablation. *Front Cardiovasc Med*; 9. Epub ahead of print 2 December 2022. DOI: 10.3389/FCVM.2022.1042573.

19. Thind M, Oraii A, Chaumont C, et al. Predictors of nonpulmonary vein triggers for atrial fibrillation: A clinical risk score. *Heart Rhythm* 2024; 21: 806–811.

20. Jud FN, Obeid S, Duru F, et al. A novel score in the prediction of rhythm outcome after ablation of atrial fibrillation: The SUCCESS score. *Anatol J Cardiol* 2019; 21: 142–149.

21. Nastasă A, Bogdan Ștefan, Iorgulescu C, et al. New Score for Predicting Results after Catheter Ablation for Atrial Fibrillation: VAT-DHF. *J Clin Med* 2024; 13: 61.

22. Williams SE, Roney CH, Connolly A, et al. OpenEP: A Cross-Platform Electroanatomic Mapping Data Format and Analysis Platform for Electrophysiology Research. *Front Physiol* 2021; 12: 160.

23. Vigneswaran V, Gharaviri ; A G, Klis ; M K, et al. Enhancing OpenEP: atrial conduction velocity and conduction velocity heterogeneity quantification through EP Workbench. *Europace*; 26, https://academic.oup.com/europace/article/26/Supplement_1/euae102.626/7681470 (2024, accessed 25 July 2024).
